# HIV drug resistance profile in South Africa: Findings and implications from the 2017 national HIV household survey

**DOI:** 10.1371/journal.pone.0241071

**Published:** 2020-11-04

**Authors:** Sizulu Moyo, Gillian Hunt, Khangelani Zuma, Mpumi Zungu, Edmore Marinda, Musawenkosi Mabaso, Vibha Kana, Monalisa Kalimashe, Johanna Ledwaba, Inbarani Naidoo, Sinovuyo Takatshana, Tebogo Matjokotja, Cheryl Dietrich, Elliot Raizes, Karidia Diallo, Gurpreet Kindra, Linnetie Mugore, Thomas Rehle

**Affiliations:** 1 Human Sciences Research Council, Pretoria, South Africa; 2 School of Public Health, University of Cape Town, Cape Town, South Africa; 3 Centre for HIV and STIs, National Institute of Communicable Diseases, Johannesburg, South Africa; 4 School of Public Health, University of the Witwatersrand, Johannesburg, South Africa; 5 Division of Global HIV and TB, Centers for Disease Control and Prevention, Pretoria, South Africa; 6 Division of Global HIV and TB, Centers for Disease Control and Prevention, Atlanta, GA, United States of America; Azienda Ospedaliera Universitaria di Perugia, ITALY

## Abstract

**Background:**

HIV drug resistance (HIVDR) testing was included in the 2017 South African national HIV household survey. We describe the prevalence of HIVDR by drug class, age, sex and antiretroviral drugs (ARV) status.

**Methods:**

Dried blood were spots tested for HIV, with Viral load (VL), exposure to ARVs and HIVDR testing among those HIV positive. HIVDR testing was conducted on samples with VL ≥1000 copies/ml using Next Generation Sequencing. Weighted percentages of HIVDR are reported.

**Results:**

697/1,105 (63%) of HIV positive samples were sequenced. HIVDR was detected in samples from 200 respondents (27.4% (95% confidence interval (CI) 22.8–32.6)). Among these 130 (18.9% (95% CI 14.8–23.8)), had resistance to non-nucleoside reverse transcriptase inhibitors (NNRTIs) only, 63 (7.8% (95% CI 5.6–10.9)) resistance to NNRTIs and nucleoside reverse transcriptase inhibitors, and 3 (0.5% (95% CI 0.1–2.1)) resistance to protease inhibitors. Sixty-five (55.7% (95% CI 42.6–67.9) of ARV-positive samples had HIVDR compared to 112 (22.8% (95% CI 17.7–28.7)), in ARV-negative samples. HIVDR was found in 75.6% (95% CI 59.2–87.3), n = 27, samples from respondents who reported ARV use but tested ARV-negative, and in 15.3% (95% CI 6.3–32.8), n = 7, respondents who reported no ARV use and tested ARV-negative. There were no significant age and sex differences in HIVDR.

**Conclusion:**

27% of virally unsuppressed respondents had HIVDR, increasing to 75% among those who had discontinued ARV. Our findings support strengthening first-line ARV regimens by including drugs with a higher resistance barrier and treatment adherence strategies, and close monitoring of HIVDR.

## Introduction

Access to antiretroviral therapy (ART) has expanded globally in the last decade, reaching 25.4 million individuals in 2017 [1]. Of the 20.7 million people living with HIV (PLHIV) in Eastern and Southern Africa in 2017, 15 million were on treatment [1]. In South Africa, more than 7 million people were living with HIV in 2017, and 4.4 million of them were receiving ART [2]. The country plans to further expand treatment coverage in line with the National Strategic Plan for HIV, Tuberculosis) (TB) and sexually transmitted infections (STIs) 2017–2022 (NSP 2017–2022) [3], which aims to achieve the 90–90–90 Joint United Nations Programme on HIV/AIDS targets [4] in every district by 2022.

Universal test and treat and reduced mortality in PLHIV [5] have meant more individuals on ART for longer periods of time, and consequently, the emergence of HIV drug resistance (HIVDR) [6]. Weaknesses in HIV treatment programmes, including high default rates, poor viral suppression levels, limited viral load (VL) testing, delayed response to high VL, and limited testing for resistance, can further increase the levels of HIVDR. HIVDR can undermine the benefits and successes of ART programmes [6, 7] and jeopardize the achievement of the 90–90–90 treatment targets, specifically VL suppression which has a direct impact on new infections [4]. The World Health Organization (WHO) therefore recommends surveillance for HIVDR through routine monitoring for early indicators of poor HIV programme performance, surveillance for pre-treatment resistance (PDR), and evaluation of resistance acquired during therapy failure [6].

Data indicates increasing levels of HIVDR across the world. A systematic review of 26 studies in low- and middle-income countries (LMIC) conducted between 2014 and 2017, found that 9.7% of adults on ART had HIVDR [7], and most prevalent amongst those on non-nucleoside reverse transcriptase inhibitor (NNRTI)-based regimens in Southern and Western African countries (>87%). Prevalence of pretreatment NNRTI resistance in in southern Africa in 2016 was estimated at 11% with a yearly increase in odds of 23% [7]. Within Southern and Central Africa, recent studies have reported PDR rates ranging from 9% to 16.3% [8–11]. A provincial surveillance system in South Africa found PDR prevalence of 11% over the period 2014–2015 [12], while sentinel surveillance estimated national TDR prevalence (NNRTI) at 5.4% [13]. In Population HIV Impact Assessment surveys (PHIAs) conducted in Malawi and Zimbabwe (respondents 15–59 years), Drug Resistant Mutations (DRMs) were detected in 4/26 (15%) and 3/30 (10%) of individuals classified as recently infected with HIV, respectively, consistent with the prevalence observed in the PDR surveys in the African Region [6].

This study presents the profile of HIVDR among PLHIV enrolled and tested in a nationally representative HIV household survey conducted in South Africa in 2017 [2].

## Methodology

### Design and sampling

The national HIV household survey was a national cross-sectional survey that included people of all ages in all provinces of South Africa, in 2017. The survey included people living in hostels while those living in educational institutions, old-age homes, hospitals, and uniformed-service barracks were excluded. All members of the selected households were invited to participate. The design was similar to that implemented in four previous HIV surveys conducted in 2002, 2005, 2008, and 2012 [14–17], as was targeted to be representative of the general population in the country. A stratified master sample of 1000 small area layers (SALs) was randomly sampled with probability proportional to size. The sample was stratified by province and locality type [urban, rural formal (farms) and rural informal (traditional rural villages)], as defined by Statistics South Africa [18]. A systematic random sample of 15 households was selected within each sampled SAL. Data collection took place between December 2016 and January 2018, and sample analysis was completed in June 2018.

### Data collection, and biomarker testing

Survey interviews and collection of blood samples were undertaken in the household after written or verbal consent (for minors, guardian consent with assent as appropriate). Effort was made to ensure confidentiality of the interview process, data transmission and processing. The questionnaires included a household questionnaire for household information and age targeted questionnaires, one for parents/guardians of children aged 0 to 11 years, one for adolescents aged 12 to 14 years, and one for those aged 15 years and older. The questionnaires included a question about daily use of ARVs.

Dried blood spot (DBS) samples were collected by finger prick (or heel prick in infants) and were tested for HIV antibodies using an algorithm with two different enzyme immunoassays (EIAs) and a nucleic acid amplification test (NAAT) for validating all positives. The HIV status of children under two years old was confirmed with the NAAT. Testing for HIV antibodies was conducted at Global Clinical and Viral laboratories in Durban South Africa, with quality assurance testing conducted at the South African Medical Council. HIV-positive (HIV+ve) specimens were further tested for: i) antiretroviral drugs (ARVs), ii) HIV VL, iii) Limiting Antigen (LAg) Avidity EIA (LAg-Avidity EIA, Portland, USA) as part of a multisassay incidence estimation algorithm that took into account ARV treatment and HIV VL [2], in samples from respondents 2 years and older, and iv) HIVDR genotyping for HIVDR.

Testing for ARVs was by High Performance Liquid Chromatography coupled with Tandem Mass Spectrometry [17], and included ARVs in first-, second-, and third-line ART in the public sector in South Africa at the time: nevirapine, efavirenz, atazanavir, darunavir, and lopinavir. The limit of detection was set at 0.02 μg/ml for each drug with a signal-to-noise ratio of at least 5:1 for all the drugs. Samples testing positive for at least one drug were classified as ARV positive (ARV+ve) and those testing negative for all drugs were classified as ARV negative (ARV-ve). Participants who reported that they were taking ARVs daily but tested negative for ARVs in the laboratory were classified as ARV defaulters. Those who reported not taking ARVs regularly and tested negative for ARVs in the laboratory we classified as potentially ARV naïve. (We had no further data to confirm this). Testing for ARVs was undertaken at the Division of Clinical Pharmacology in the Department of Medicine at the University of Cape Town, South Africa. At the time of our survey most patients on ARVs were on fixed-dose combination therapy such as TDF/FTC/EFV (TEE) or TDF/3TC/EFV (TLE). By selecting Efavirenz as the NNRTI ‘backbone’ drug of these combinations we did not need to test for the other NRTI companion drugs, Tenofovir, Emtricitabine or Lamivudine respectively.

VL testing used the Abbott platform (Abbott m2000 HIV Real-Time System, Abbott Molecular Inc., Des Plaines, IL, USA), and was undertaken at the National Institute for Communicable Diseases (NICD) in Johannesburg South Africa. Samples with VL ≥ 1000 copies/ml were classified as virally unsuppressed and were submitted for HIVDR testing.

HIVDR testing was undertaken at the NICD and was conducted by Next Generation Sequencing (NGS). DBS were excised, immersed in 2 ml of NucliSENS lysis buffer (BioMerieux, Nürtingen, Germany) and lysed on a roller mixer at room temperature. Total nucleic acid was extracted using the NucliSENS EasyMAG® automated system according to the manufacturer’s instructions. Amplification of a 1,084 base pair polymerase chain reaction (PCR) fragment consisting of codons 1–99 of protease and codons 1–250 of reverse transcriptase was performed as previously described [19], with the exception of 400 μM of each primer that was used for reverse transcription (RT) polymerase chain reaction (PCR). PCR products were sequenced on the Illumina MiSeq using MiSeq Reagent Kit v3 (Illumina Inc San Diego, CA, USA). FastQ files generated by the Illumina MiSeq were analysed for mutations associated with resistance to ART using Geneious vR9, based on the Stanford v8.0 algorithm and using a 10% prevalence detection threshold. All specimens with a sequence similarity of <1.0% were repeated from extraction for confirmatory purposes [20]. HIVDR mutations were graded using the 3-level grading approach of susceptible, intermediate and high-level resistance using the 2017 update of the Drug Resistance Mutations in HIV-1 list as the reference [21].

### Data analysis

Data from samples tested for HIVDR are presented. Survey weights were applied to generate nationally representative estimates, and the analyses took into account the multi-level stratified design of the survey. The final individual weights were benchmarked against the 2017 mid-year population estimates by age, race, age, and province [2]. Weighted data were analysed using STATA version 15 (Stata Corporation, College Station, TX, USA). Estimates are presented as weighted percentages with 95% confidence interval (CI). The Pearson’s Chi Square test was used to compare categorical variables, with p < 0.05 used to determine statistical significance. The median and interquartile range were used to describe continuous variables.

### Ethics

The survey was approved by the Human Sciences Research Council (HSRC) Research Ethics Committee (REC: 4/18/11/15). The study was also reviewed in accordance with the Centers for Disease Control and Prevention (CDC) human research protection procedures and was determined to be research. CDC investigators did not interact with human subjects or have access to identifiable data or specimens for research purposes (CGH 2016-143a).

## Results

Samples from 23,836 participants were tested for HIV and among these 2,994 (median age 36 years (interquartile range (IQR) 26–48) tested HIV+ve translating to an estimated HIV prevalence of 14.0% (95% CI 13.1–15.0) [2]. The testing response rate for the entire survey was 61.8% [2]. The survey data were representative of the HIV population in South Africa, with women more likely to provide a blood sample for testing that males [2].

**Fig 1:**
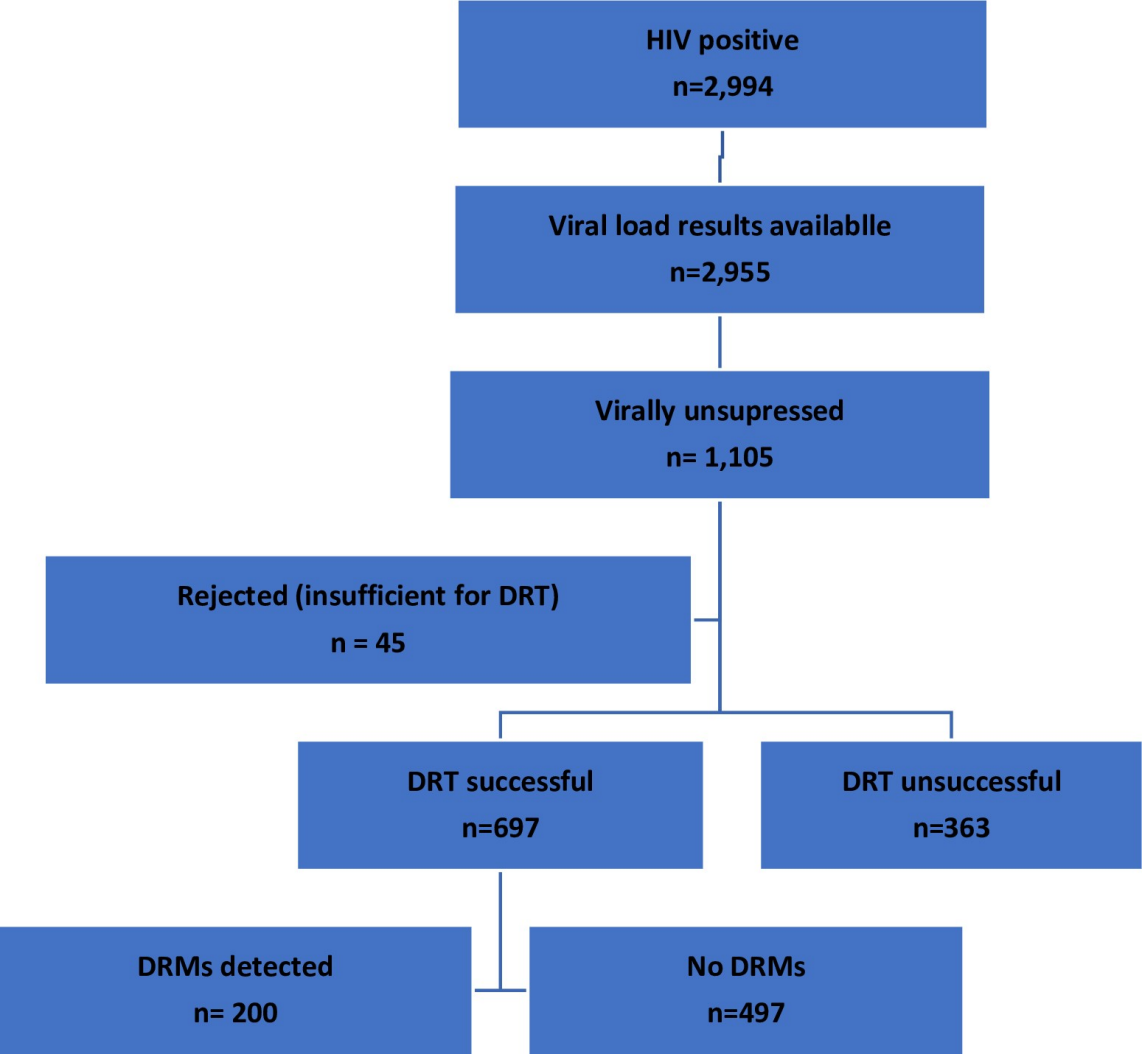
Samples tested for HIV drug resistance, South Africa, 2017. DRT- Drug Resistant Testing; DRMs- Drug Resistant Mutations.

Among the HIV+ve samples with VL results (n = 2,955), 1,105 (37.4%) were virally unsuppressed and among these, 697 (63.1%) were successfully genotyped (see Fig 1), median age 34 years (IQR 27–44). Among those who were virally unsuppressed ARV laboratory data was available for 971 among whom 743 (76.5%) tested ARV negative, and among these 62(8.3%) respondents had reported that they taking ARVs and were defined as ARV defaulters.

Table 1 shows the characteristics of respondents who provided samples that were successfully genotyped and those that were not. There was no difference in the two groups by sex and locality type. There were significant differences by age and ARV status with a greater proportion of samples from children age 0–14 years among those that were not successfully genotyped, and a greater proportion of samples that tested ART negative that were successfully genotyped.

**Table 1:** Characteristics of respondents with samples that were successfully genotyped and those that were not.

	DRT successful n = 697	DRT unsuccessful n = 363	
	% (95% CI)	% (95% CI)	p value
**Sex**			
Male	69.3 [62.2–75.7]	30.7[24.3–37.8]	0.141
Female	63.1[57.8–68.1]	36.9 [31.9–42.2]	
**Age category (years)**			
0–14	35.6 [22.8–50.8]	64.4 [49.2–77.2]	<0.001
15–24	66.3 [54.6–76.3]	33.7 [23.7–45.4]	
25+	68.2 [63.2–72.7]	31.8 [27.3–36.8]	
**ARV status**			
ARV–ve	70.8 [65.8–75.4]	29.2 [24.6–34.2]	P<0.001
ARV +ve	45.7 [37.0–54.6]	54.3 [45.4–63.0]	
**Locality type**			
Urban	66.6[60.9–71.9]	33.4 [28.1–39.1]	0.436
Rural/informal (tribal areas)	61.9 [54.1–69.1]	38.1 [30.9–45.9]	
Rural (farms)	72.8 [53.7–86.1]	27.2 [13.9–46.3]	

# Weighted percentages; ARV- Antiretrovirals.

### Overall HIV drug resistance

**Fig 2:**
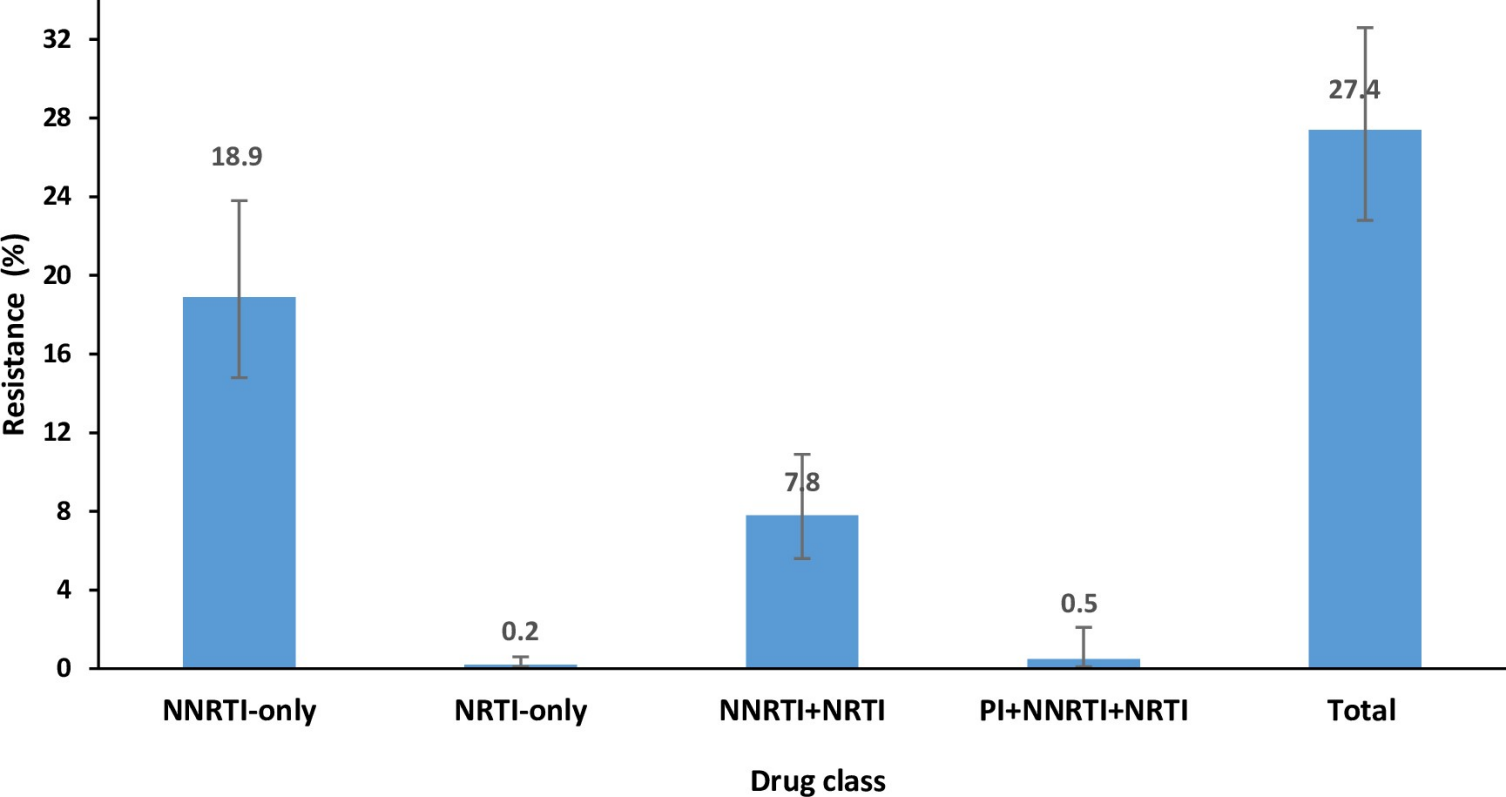
HIV drug resistance among virally unsuppressed survey respondents, South Africa, 2017. NNRTI- non-nucleoside reverse transcriptase inhibitors; NRTI- nucleoside reverse transcriptase inhibitors; PI-Protease inhibitors. Bars represent the 95% Confidence interval.

Resistance was identified in 200/697 samples, giving a prevalence of 27.4% (95% CI 22.8––32.6). Median VL in these samples was 18,913 (IQR 6,150–73,221). The most frequent mutation by drug class was resistance to NNRTIs only, found in 18.9% (95% CI 14.8–23.8), n = 130 of samples (Fig 2). Resistance to nucleoside reverse transcriptase inhibitors (NRTIs) was in 0.2% (95% CI 0.1–0.6), n = 4 samples. Dual resistance to NNRTIs and NRTIs was in 7.8% (95% CI 5.6–10.9), n = 63 samples, while resistance to three drug classes NNRTIs, NRTIs, and protease inhibitors (PI) was found in 0.5% (95% CI 0.1–2.1), n = 3 samples.

### HIV drug resistance by demographic characteristics

Overall, resistance was detected in 29.4% of samples from males and in 25.8% of samples from females, p = 0.473 (Table 2). There was no significant difference between NNRTI-only and dual NNRTI and NRTI resistant samples by sex overall. A third (33.7%) of samples from respondents younger than 14 years had resistance, compared to 30.5% in samples for the youth (15–24 years) and 26.6% from those 25 years and older, p = 0.684. There were no differences in the proportion of respondents with resistance by sex in the different age categories: children younger than 14 years (p = 0.823), youth aged 15–24 years (p = 0.067), those in the reproductive age group (15–49 years) (p = 0.676), and those 50 years and older (p = 0.191). There were also no differences by drug class in these age and sex strata (Table 2).

**Table 2:** HIV drug resistance by sex, age, and locality type, South Africa, 2017.

Variable	n	Resistance %# (95% CI)	p value	NNRTI only resistance %# (95% CI)	p value	Dual NNRTI & NRTI resistance %# (95% CI)	p value
**Sex**							
Male	252	29.4 (22.5–37.4)	0.473	19.6 (13.5–27.7)	0.772	9.7 (5.8–15.7)	0.202
Female	445	25.8 (19.8–32.8)		18.3 (13.2–24.8)		6.3 (4.2–9.5)	
**Age**							
0–14 years (Total)	26	33.7 (17.6–54.7)		17.7 (7.2–37.4)		14.9 (5.3–35.2)	
Male	12	35.8 (14.6–64.6)	0.823	**	0.749	**	0.461
Female	14	31.5 (11.1–62.8)		**		**	
15–24 years (Total)	98	30.5 (18.7–45.5)		22.1 (12.6–35.9)		5.7 (1.7–16.8)	
Male	25	48.7 (26.7–71.2)	0.067	36.7 (17.8–60.7)	0.063	**	**
Female	73	22.6 (11.4–39.7)		15.8 (7.4–30.7)		**	
25+ years (Total)	573	26.6 (21.7–32.2)		18.4(14.0–23.8)		7.9 (5.4–11.4)	
Male	215	27.1 (19.6–36.1)	0.89	18.0 (11.5–27.0)	0.88	9.1(5.1–15.8)	0.45
Female	358	26.3 (19.6–34.1)		18.8 (13.1–26.3)		6.9 (4.3–10.8)	
15–49 years (Total)	568	27.5 (22.5–33.2)		19.2 (14.8–24.4)		7.8(5.3–11.3)	
Male	207	28.8 (21.4–37.6)	0.676	19.7 (13.2–28.2)	0.857	9.1 (5.1–15.9)	0.392
Female	361	26.4 (19.7–34.4)		18.7 (13.0–26.2)		6.6 (4.1–10.4)	
50+years (Total)	103	24.1 (14.8–36.7)		17.0 (8.9–30.0)		5.7 (2.5–12.8)	
Male	33	34.8 (15.1–61.5)	0.191	21.8 (5.8–56.0)	0.562	**	**
Female	70	19.8 (11.7–31.4)		15.1 (8.2–26.2)		**	
**Locality type**							
Urban	388	29.5 (23.5–36.4)	0.077	21.0 (15.6–27.6)	0.138	7.6 (4.8–11.9)	0.792
Rural informal (tribal areas) & rural formal (farms)	309	23.1 (17.4–30.1)		14.6 (9.8–21.1)		(5.2–12.9)	

# Weighted percentages.

** Sample was too small for the respective disaggregation and comparisons.

NNRTI- non-nucleoside reverse transcriptase inhibitors; NRTI- nucleoside reverse transcriptase inhibitors.

Resistance was detected in 29.5% of samples from respondents in urban areas, and in 23.1%, p = 0.077 of samples from rural areas, Table 2. There were no statistically significant differences in the proportion of samples with resistance by drug class between the locality types.

### HIV drug resistance by ARV status

Of the 200 samples with HIVDR, 177 had laboratory results for exposure to ARVs, with (65) 29.4% ARV+ve. HIVDR was found in 55.7% of the ARV+ve samples compared to 22.8% in ARV-ve samples p <0.001 (Table 3). When stratified by drug class, 14.3% (95% CI 7.5–25.6) of those who tested ARV+ve had resistance to NNRTIs only compared to 20.0% (95% CI 15.4–25.7) of those who tested ARV-ve, p = 0.311. Dual NNRTI and NRTI resistance was found in 40.4% (95% CI 29.6–52.2) of those who were ARV+ve and in 2.1% (95% CI 0.6–6.8) of those ARV-ve, p<0.001.

**Table 3:** HIV drug resistance by ARV status, South Africa, 2017.

Variable	n	Resistance %# (95% CI)	p value	NNRTI-only resistance %# (95% CI)	p value	Dual NNRTI & NRTI resistance %# (95% CI)	p value
ARV+ve (laboratory confirmed)	102	55.7 (42.6–67.9)	<0.001	14.3 (7.5–25.6)	0.311	40.4 (29.6–52.2)	<0.001
ARV–ve (laboratory confirmed)	517	22.8 (17.7–28.7)		20.0 (15.4–25.7)		2.1 (0.6–6.8)	
On ARVs by self-report and tested negative for ARVs in the laboratory (ART defaulters)	41	75.9 (59.2–87.3)	<0.001	56.4 (34.4–76.2)	<0.001	14.3 (2.5–52.1)	
Not on ARVs by self-report and tested negative for ARVs in the laboratory	33	15.3 (6.3–32.8)		15.3 (6.3–32.8)		0	

# Weighted percentages.

ARV- Antiretrovirals; NNRTI- non-nucleoside reverse transcriptase inhibitors; NRTI- nucleoside reverse transcriptase inhibitors.

### HIVDR by reported ARV use

Ninety-four samples tested for HIVDR were from respondents who reported daily ARV use at the time of the survey (median age 33 years, IQR 31–45, 53.1% females). Those who reported taking ARVs, but tested ARV-ve were classified as ART defaulters, (n = 41, median age 33 years, IQR 28–38, 56.5% females). HIVDR was detected in 75.9% of these samples, (Table 3); 56.4% of the samples had NNRTI-only resistance (95% CI 34.4–76.2) and 14.3% (95% CI 2.5–52.1) had dual NNRT and NRTI resistance. The sample was too small to estimate resistance to PI-containing regimens.

Thirty-three samples tested for HIVDR were from respondents who reported they were not taking ARVs and tested ARV-ve in the laboratory (median age 38 years, IQR 28–47, and 72.9% were female). HIVDR was detected in 15.3% (95% CI 6.3–32.8) of these samples and all had NNRTI-only resistance. The proportion of HIVDR in this group was significantly lower than in respondents who were taking ARVs, 15.3% vs 55.7% p = 0.004.

### HIV drug resistance among respondents recently infected with HIV

HIVDR was detected in 7of 32 samples (21.8% unweighted percentage) recently infected with HIV, and with genotype data available. All seven samples had resistance to NNRTIs (Table 4) and were not on ARVs as our incidence algorithm excluded those on ART [2].

**Table 4:** HIV drug resistance among respondents classified as recently infected with HIV, South Africa, 2017.

Age	Sex	Mutation type
18	Male	K103N
22	Female	K103N, P225H
34	Male	K103N
32	Female	K103S
25	Female	K103N
46	Female	K103N
61	Female	K103N

## Discussion

With the largest public sector ART programme in the world, monitoring of population level HIVDR is critical in South Africa since HIVDR levels can impact achievement of the UNAIDS 90–90–90 targets [22]. The HIVDR estimates from this nationally representative HIV survey that were determined by NGS provide useful HIVDR data for all PLHIV in the country. The sample includes virally unsuppressed individuals who may have not yet accessed care, those who may have been disengaged from care (treatment interrupters and defaulters from ART), and those who may have been accessing care from the private sector in addition to those within the public sector ART programme. The data thus represent a comprehensive picture of HIVDR among PLHIV in South Africa.

HIVDR levels were high. Resistance was detected in more than a quarter (27.4%) of samples from virally unsuppressed respondents. Nearly a fifth of the respondents had resistance to NNRTIs. This is consistent with the lower genetic barrier of NNRTIs to resistance [23]. The high HIVDR levels are significant since HIVDR is associated with increased mortality [24, 25] and lower effectiveness of treatment regimens [6], and could severely impact attainment of viral suppression levels needed to end the HIV epidemic [4]. Furthermore, high levels of HIVDR among those on ART (55.7%) highlights a risk of transmission of resistant HIV especially given that in this survey only 28.1% of respondents reported consistent use of condoms [2].

We found low levels of resistance to second-line therapy which includes PIs, and this could indicate appropriate limited prescription of second-line therapy, since PI mutations are much less common than NNRTI and NRTI mutations because PIs have a much higher genetic barrier to resistance [26]. However, limited use of second-line could also be due to other reasons, including lower than ideal levels of VL testing [27] (since levels of VL are key in informing decisions to switch to second-line therapy), poor tolerability, higher cost, and concerns about more frequent dosing in second-line regimens [28]. Analysis of laboratory VL tests undertaken in South Africa between April 2014 and March 2015 showed that 25% of ART clients who were eligible for a VL test during that period had not been tested [27]. In addition, while 78% of ART clients were virally suppressed, only 58% were known to be suppressed by healthcare workers indicating that VL results were not always used to monitor the response to ART [27].

We found NNRTI-monoresistance in samples from 18.9% respondents, and this was the most frequent resistance type as expected in settings where NNRTIs are a key part of first-line treatment regimens. NNRTI mono-resistance also occurred in 15.3% of samples from respondents who tested ARV–ve and reported not taking ARVs. These findings further indicate the low resistance barrier of efavirenz, the most widely used NNRTI in South Africa. Mbuagbaw et al [29] showed that people with pretreatment NNRTI resistance who then receive NNRTI regimens were significantly more likely to experience viral failure or death and were also more likely to discontinue treatment. These findings indicate the importance of regimens that do not contain NNRTIs [6, 30, 31].

HIVDR were most prevalent in samples from respondents with prior exposure to ART (those who reported taking ARVs but tested ARV-ve in the laboratory and were classified as ART defaulters):– 75.9% had DRMs, 56.4% of them with NNRTI mono-resistance. This estimate is much higher than previously reported in South Africa, where HIVDR was found in 37.5% of samples of patients with prior exposure to ART who were enrolled as part of sentinel surveillance in public clinics in three provinces [32]. This difference (75.9% vs 37.5%) could be due to the different populations in these two analyses: household survey vs facility recruitment, or the relatively small sample size in the survey. NNRTI resistance was found in 18.6% of samples in sentinel surveillance compared to 56.4% in the survey, in individuals with prior exposure to ART. Other PDR surveys conducted between 2014 and 2016 in Africa also found that PDR was driven by NNRTI resistance with NNRTI PDR estimates of 17.5% (95% CI 2.3–65.2) and 34.8% (95% CI 25.2–45.8) in Uganda and Namibia, respectively [6].

Our findings of high HIVDR levels among people with prior ART exposure compared to those who reported not taking ARVs and tested ARV-ve are consistent with previous reports that PDR is more than two-fold higher among people starting first-line ART with prior ARV drug exposure, compared to ARV drug-naive individuals [6, 33]. The findings also highlight the impact of poor retention of people in ART care, and the impact on resistance to treatment.

Our estimated HIVDR of 55.7% among virally unsuppressed people on ART is lower than reported from facility-based studies in South Africa. Hunt et al, 2017 [34] found DRMs in 84% and 89% of HIV+ve virally unsuppressed samples from people who had been on ARVs for 12–15 months and 24–36 months respectively in KwaZulu-Natal (KZN), South Africa. Other studies conducted between 2006 and 2012, also in KZN, have reported DRM levels ranging from 83.5% to >97% among people failing therapy [35–37]. These discrepancies could be due to the different populations analysed, as facility-based studies more likely to sample PLHIV with clinical symptoms that may correlate with failing therapy and declining CD4 counts, while the survey samples those with more varied adherence and clinical engagement patterns.

Although the numbers are small our finding of 7 samples with DRMs out of 32 samples from respondents recently infected with HIV (21.8%) could suggest higher levels of transmitted HIVDR than found in studies in KZN [38–40]. It is also higher than findings from PHIAs conducted, where there were 4/26-15.4% (Malawi) and 3/30-10.0% (Zambia) samples with DRMs from infected samples among adults aged 15–59 years [6].

Rates of successful PCR amplification from dried blood spots were lower than expected. The WHO recommends that DBS specimens with a viral load of >1,000copies/ml be used for HIVDR testing, but acknowledges poor amplification rates at lower VL levels. As we tested all specimens with VL>1,000 copies/ml, this could have contributed to lower overall PCR non-amplification rates [41]. In addition, specimens are maintained at ambient temperature for >2 weeks have increased levels of PCR non-amplification. As we were using two DBS cards for all tests in for this survey, the cards were used for a series other laboratory tests before one became available for HIVDR testing. These successive periods at ambient temperature coupled with freeze-thaw cycles could have contributed to overall specimen degradation [42]

This is the first analysis of HIVDR in a nationally-representative sample of PLHIV in South Africa, and therefore includes PLHIV who are not in care, and those accessing care from the private sector who would be excluded from HIVDR surveillance that utilizes data from public health facilities as recommended by WHO [43]. However, there were some limitations. About a third of virally unsuppressed samples could not be successfully genotyped. Some population sub-groups (e.g. prior ART exposure) are not entirely comparable to those described in the WHO HIVDR reports given the survey approach to data collection. Finally, the sample of incident cases was small, although it was comparable to samples in PHIA surveys conducted in Malawi and Zambia.

## Conclusions

Analysis of a nationally representative sample of PLHIV in South Africa, found high levels of resistance to first-line ART regimens among virally unsuppressed PLHIV. HIVDR among people who have defaulted from ART are particularly high. Our findings support strengthening first-line ARV regimens by including drugs with a higher resistance barrier, and treatment adherence strategies, and close monitoring of HIVDR.
